# Associations between digital dermatitis lesion grades in dairy cattle and the quantities of four *Treponema* species

**DOI:** 10.1186/s13567-018-0605-z

**Published:** 2018-10-29

**Authors:** Caroline Beninger, Syed Ali Naqvi, Sohail Naushad, Karin Orsel, Chris Luby, Hooman Derakhshani, Ehsan Khafipour, Jeroen De Buck

**Affiliations:** 1 Department of Production Animal Health, Faculty of Veterinary Medicine, University of Calgary, Calgary, AB Canada; 2 Department of Large Animal Clinical Sciences, Western College of Veterinary Medicine, University of Saskatchewan, Saskatoon, SK Canada; 3 Department of Animal Science, Faculty of Agricultural and Food Sciences, University of Manitoba, Winnipeg, MB Canada

## Abstract

Digital dermatitis (DD) presents as painful, ulcerative or proliferative lesions that lead to bovine lameness affecting economic efficiency and animal welfare. Although DD etiological agent(s) have not been established, it is widely accepted that DD is a polymicrobial disease significantly associated with species of *Treponema* and the non-linear disease progression may be attributed to interactions among infecting bacteria. We postulated the morphological changes associated with DD lesion grades are related to interactions among infecting species of *Treponema*. We developed a novel species-specific qPCR that can identify the absolute abundance of the four of the most common species of *Treponema* in DD, *T. phagedenis*, *T. medium*, *T. pedis* and *T. denticola*, in a single reaction. We found species abundance and the number of distinct *Treponema* species present is higher in active, ulcerative lesions than in healing lesions, chronic lesions, and DD-free skin*. Treponema* spp. were present in both DD-free skin and M3 lesions following treatment with oxytetracycline. We have also found positive correlations among *T. phagedenis*, *T. medium* and *T. pedis* indicating they are significantly more likely to be found together than apart and their absolute quantities tend to increase together, a relationship which is not present with *T. denticola*. Further, we found *Treponema*, particularly viable *T. denticola*, in lesions 5 days post treatment with oxytetracycline (M3). Our findings suggest that pathogenicity may be closely associated with *Treponema* abundance, particularly *T. phagedenis*, *T. medium* and *T. pedis*, and interactions among them, independent of *T. denticola*. Our results provide a novel, consistent method to identify species of *Treponema* within DD lesions and associate *Treponema* spp. and abundance with morphological changes related to host pathogenicity.

**Electronic supplementary material:**

The online version of this article (10.1186/s13567-018-0605-z) contains supplementary material, which is available to authorized users.

## Introduction

Globally, digital dermatitis (DD) is a leading cause of infectious lameness in dairy cattle [1]. Digital dermatitis is mainly found on the plantar aspect of a cow’s foot, presenting as an ulcerative or proliferative lesion along the coronary band, affecting the area between the heel bulbs [1]. A review paper on lameness prevalence studies throughout the world between 1993 and 2014 found that between 25 and 55% of dairy cattle are clinically lame on average, 15–22% of cows have one or more DD lesions, and up to 94% of herds have DD [2–4]. The infectious, ulcerative or proliferative lesions caused by DD contribute to animal welfare concerns and significant economic losses due to premature culling, milk loss, decreased fertility, and treatment costs [4, 5]. The complex polymicrobial nature of the disease and non-linear lesion progression have contributed to the inability to determine the etiological agent(s) for DD despite significant economic incentives [6, 7]. Species of *Treponema,* fastidious, anaerobic bacteria, are consistently isolated from DD lesions in polytreponemal communities [8–10]. While there is considerable evidence implicating *Treponema* spp. as causative agents in DD, Koch’s postulates have yet to be satisfied [1, 10–12]. It has been more than 40 years since DD was first described, and despite efforts to prevent the disease with improved hygiene practices, regular footbaths, and bedding modifications, effective treatments that eliminate the disease from herds have not been identified [5, 13]. Furthermore, treatments for DD with 100% sustained cure rate have not been identified and treated lesions are prone to relapsing into active disease states or remaining chronic [14–16].

Methods to simultaneously identify multiple species of *Treponema* within DD lesions are currently limited to 16S rRNA gene sequencing-based microbiome approaches which cannot reliably resolve species composition due to partial 16S gene coverage, limited complete genomes available and many unidentified *Treponema* species. *Treponema* phylotypes are based on culture independent methods such as 16S rRNA and/or *flaB*2 sequencing and 16S–23S intergenic spacer region sequence analysis which distinguishes phylotypes based on size [9, 15, 17, 18]. Phylotypes are defined as clusters of *Treponema* in which the 16S rRNA sequence differs by approximately 2% from a known *Treponema* spp. and species within a cluster must share ≥ 99% sequence similarity with other members of their cluster [1]. Phylotypes are mostly named based upon their sequence similarity to human *Treponema* isolates, primarily periodontal disease isolates [10]. Because researchers use different sequences for clustering isolates are clustered differently, impacting prevalence data between studies [9, 15, 17, 18].

Previous studies have associated *Treponema* abundance with lesion severity and temporal progression, demonstrating that the composition of species present changes considerably as lesions progress [19]. The microbial community present within DD lesions has been shown to correlate with morphological changes of lesions; however, while this relationship is significantly correlated with the 5 grade Iowa DD scoring system, which primarily differs from M-grade scoring by having a temporal aspect to lesion progression and adding 2 early grade lesions, it has not been examined using the M-scoring system [19]. Therefore, current evidence suggests *Treponema* spp. and bacterial community composition may be related to morphological changes associated with grades of DD lesion grades.

There is increasing evidence implicating multiple species of *Treponema* in DD etiology and pathogenicity; however, there is a knowledge deficit regarding potential interactions among species present [20]. Based on current evidence, we postulate a relationship between the *Treponema* spp. present and DD lesion grades and/or DD progression that may be facilitated by synergistic interactions among species. The aims of this study were to examine the correlations among *Treponema* spp. within and between lesion grades. For this purpose, a novel species-specific qPCR was developed to identify species present, their abundance, and relative proportions in DD lesions. Identifying correlations between species of *Treponema* present in DD lesions may provide essential targets for vaccines and treatment development by indicating species synergies required for enhanced virulence or pathogenicity.

## Materials and methods

### Farm and animal details

Farms were selected based on previous DD prevalence information and geographical location, facilitated by two certified Alberta hoof trimmers. Samples were collected from 10 Alberta farms and one abattoir between Calgary and Ponoka throughout a 1.5-year period. Additionally, 10 samples were collected from a Saskatchewan dairy herd that has been a closed farm for over 50 years with a reported zero DD prevalence (Rayner Dairy Research and Teaching Facility, University of Saskatchewan). All animal use was approved under protocol #AC16-0070, by the University of Calgary Veterinary Services Animal Care Committee (VSACC) under the guidance of the Canadian Council on Animal Care (CCAC) prior to the onset of the study.

### DD scoring

Digital dermatitis lesions were identified on farm in the trimming chute according to the M-grade scoring system and limited to lesions located between the heel bulbs [13, 21]. Briefly, M1 and M2 lesions were ulcerative masses along the coronary band, distinguished by a lesion smaller or larger than 2 cm, respectively [21]. Due to the difficulty in characterizing M3 lesions without treatment history, we classified M3 lesions as active lesions approximately 5 days after antibiotic treatment. M4 lesions were raised, hyperkeratotic, and proliferative, with papilliform projections and M4.1 lesions were M4 lesions with an active ulcerative area.

### Biopsy

Identified lesions were scrubbed twice using a chlorohexidine scrub followed by a 70% ethanol wipe while animals were in a no-tilt trimming chute to remove debris and clean the area. Lidocaine Neat (2–3 mL) (Lidocaine HCl 2%, DIN 00712884, Zoetis Canada Inc., Kirkland, QC, Canada) was administered subcutaneously with a 20 g needle. Small, 4 mm biopsy punches with a maximum coring depth of 7 mm (Standard Disposable Biopsy Punch, Miltex, Integra Life Sciences Corporation, York, PA, USA) were taken from various DD lesion grades from dairy cows on 10 Alberta farms and DD-free feet from the abattoir and 1 closed herd in Saskatchewan (*N* = 142). For lesions with an ulcerative focus, M1, M2, and M4.1, the biopsies were collected from the active area. “M3 biopsies” were collected from M2 lesions, identified, treated and wrapped by a certified hoof trimmer, 5 days after antibiotic treatment with soluble tetracycline250 powder (250 mg/g) (DIN 0052777, Vetoquinol, Lavaltrie, QC, Canada). Samples between the heel bulbs from non-infected hooves (*N* = 21) were collected from culled Holstein cows at the abattoir less than 2 h post-mortem. Two biopsies from DD-infected feet at the abattoir were taken to ensure *Treponema* could be cultured from tissue samples collected post-mortem, if present. Biopsies were immediately placed into semi-solid anaerobic transport media (ATM) (Anaerobe Systems, Morgan Hill, CA, USA) with the interior portion inserted first to avoid contaminating the biopsies with microorganisms from the outer skin and transported at room temperature. The transport medium, ATM, contains sodium thioglycolate and cysteine reducing agents to sustain viability of anaerobic microbes during transport with minimal multiplication. Biopsy samples from within Alberta were transported to the lab and processed within 8 h of sampling and biopsies from Saskatchewan were processed within 24 h of sampling.

### Culture and isolation

In the anaerobic cabinet (Bactron3000, Sheldon Manufacturing, Inc., Cornelius, OR, USA) (25% CO_2_, 5% H_2_, balance nitrogen), on a sterile petri dish with sterile tissue forceps and a no. 10 scalpel blade, the outermost portion of the epidermis was removed from the biopsies and discarded. The remaining interior portion was sectioned longitudinally into 4 fragments of approximately equal size and macerated. Biopsy fragments for DNA extraction and storage were inoculated into Tris–EDTA buffer (TE) (pH = 8.0) and OTEB + 20% glycerol, and stored at −20 °C and −80 °C, respectively. Biopsy fragments for culture were inoculated into oral treponeme enrichment broth (OTEB) (Anaerobe Systems, Morgan Hill, CA, USA) with 5% enrofloxacin (≥ 98% HPLC, powder, Sigma-Aldrich, Burlington, MA, USA) dissolved in DMSO, 10% rifampicin (≥ 97% HPLC, powder, Sigma-Aldrich, Burlington, MA, USA) dissolved in DMSO, and 10% equal parts bovine and rabbit serum (Gibco, Life Technologies, New Zealand and USA, respectively) (OTEBSER) and MTGE media supplemented with 5% enrofloxacin and 10% rifampicin by spread plating (MTGEER) (Anaerobe Systems, Morgan Hill, CA, USA). Biopsy fragments were inserted into MTGE medium using a no. 10 scalpel blade and sterile forceps to ensure the fragments were laterally and distally surrounded by agar. MTGEER plates were wrapped in parafilm and incubated for 7 days at 37 °C. Motile bacteria (those that migrate away from the biopsy) forming white-translucent, small, and irregular colony morphologies on the surface of, or embedded within, the agar were selected from each plate and subcultured into 1 mL of OTEBSER in 1.5 mL Eppendorf tubes and incubated for up to 10 days at 37 °C in the anaerobic cabinet. Subculture purity, absence of contaminating non-treponeme bacteria, was assessed using dark field or differential interference contrast (DIC) microscopy based on *Treponema* morphological characteristics (thin corkscrew-shaped bacteria between 0.1 and 0.4 μm in width and 4–15 μm in length, and rotational movement about the longitudinal axis) [22]. Subcultures were screened for *T. phagedenis*, *T. medium*, *T. pedis* and *T. denticola* with an in-house species-specific multiplex PCR outlined below. Cultures containing other bacteria in addition to *Treponema* were spread plated onto MTGEER, incubated for 7 days at 37 °C in the anaerobic cabinet, and subcultured into OTEBSER as above until contaminant free *Treponema* cultures were obtained.

Macerated biopsy fragments in 200 μL of TE were weighed twice and averaged prior to freezing and DNA extraction. DNA from macerated biopsy fragments in TE was extracted using DNeasy Blood and Tissue Extraction Kit according to the manufacturer’s recommendations (Qiagen, Hilden, Germany). DNA was eluted into nuclease-free ultra pure water and stored at −20 °C.

### qPCR design and optimization

A 4-plex *Treponema* species-specific qPCR was designed to quantify four *Treponema* spp. and their absolute quantities in bovine tissue samples. Genomic DNA extracted from 7 DD *Treponema* isolates, which were identified as *T. phagedenis*, *T. pedis*, and *T. medium*, from the University of Wisconsin, provided by Dr. Döpfer, were sequenced, assembled and annotated as described previously [23]. For species-specific gene identification, an in-house database containing newly sequenced *Treponema* genomes and representative genome sequences from all known bacterial species was constructed from NCBI, EZBioCloud, PATRIC, and JGI-IMG [24–27]. Potential species-specific genes were identified according to Naushad et al. [28]. Briefly, BLASTn searches were conducted on all open reading rrames (ORFs) from *T. pedis*, *T. phagedenis*, *T. medium* and *T. denticola* against the in-house database. The ORFs which were detected in a single *Treponema* species and were not found in any other representative bacterial species were considered potential species-specific genes. The specificity of potential species-specific genes was validated after blast searching against the NCBI nr database. The ORFs detected in all known strains of each species were considered unique genes. Unique genes selected for each *Treponema* species were CP004120 for *T. pedis*, WP_002698807.1 for *T. phagedenis*, WP_016523385.1 for *T. medium*, and EGC77593.1 for *T. denticola.* Based on BLAST results and available complete reference genomes for the four *Treponema* species, we assume there is a single species-specific gene copy found in each species. Primer and probes against species-specific genes were designed with idtDNA PrimerQuest software (Integrated DNA Technologies, 2017). Primer sequences were designed with an equal melting temperature (T_m_) of approximately 60 °C and probes had a Tm of approximately 70 °C or 10 °C above the primer Tm. The length of the qPCR products was set between 90 and 150 bp. Runs of consecutive nucleotides and GC clamps were avoided, and GC content was below 60% in the primers and probes (TaqMan Probe Design, Premier Biosoft). Primer and probe sequences that met the above criteria were analyzed for hairpins, self-dimers, and hetero-dimers using PriDimerCheking 0.1.0 software [29] to avoid secondary structure between primers and probes. Only sequences without hairpins at the Tm of the sequence and ∆G values, which indicate energetic favourability of a given structural conformation, more positive than −9 kcal/mol were selected.

Conventional PCR using the genes identified above were designed to verify species composition to validate the qPCR and culture composition (Table 1). Final forward and reverse primer concentrations were 0.3 μM in 23 μL of TopTaq mastermix (12.5 μL/reaction), nuclease free water, (6.5 μL/reactions) and coral load (2.5 μL/reaction) (TopTaq™ Master Mix Kit, Qiagen) and 2 μL of template DNA. Reaction conditions were 95 °C for 5 min, (94 °C 30 s, 57 °C for 30 s 72 °C 40 s) × 35, 72 °C 5 min.

**Table 1:** **Primer and probe sequences for** ***Treponema*** **species-specific fourplex qPCR and conventional PCR**

Species	Forward primer (sense)	Probe (sense)	Reverse Primer (antisense)	Size (bp)
*T. denticola* ^a^	GGAAACTTAGGAATTCGATATGTAG	AGCATACAGCGATTATAACAAAGCCCTCGA	CCTTCTTTAGTTTCTTTGTGAGG	113
*T. medium* ^a^	AAAGCGCTACGAATCCTAAG	TGCACCCTTGTTTACTACTGCACAGCC	ATCATTACCCGTCCACAAAG	119
*T. phagedenis* ^a^	CCCGCAGGAAGGTATAATC	AATCCGCCTACGACTGCGATACCA	CACAGCTGTTGTGGTATTAAG	90
*T. pedis* ^a^	ACACCGATTGTACTGAATGA	ACTACACGTGGAGTACCGAATGCT	CCACGAGCTTTCTACAGATT	118
*T. denticola* ^b^	AGGAATGGCCTTTGAACCCGCA		CCGATGAACCCGTATCTTCACCGA	458
*T. medium* ^b^	GGAACAGGCAGCCGCATTGGAT		CCGCCCATGTGAGGCTTGTGAT	515
*T. phagedenis* ^b^	TCCGCCTACGACTGCGATACCA		CGGAACTGTCACAACTGGCGGA	785
*T. pedis* ^b^	TGGATGTTACGGAAGAGACACCGA		TGCCCCACTCTTACAAGTTCATCCCA	295

^a^Primer and probe sequences for species of *Treponema* for qPCR.

^b^Conventional PCR primer sequences for *Treponema* species.

**Figure 1:**
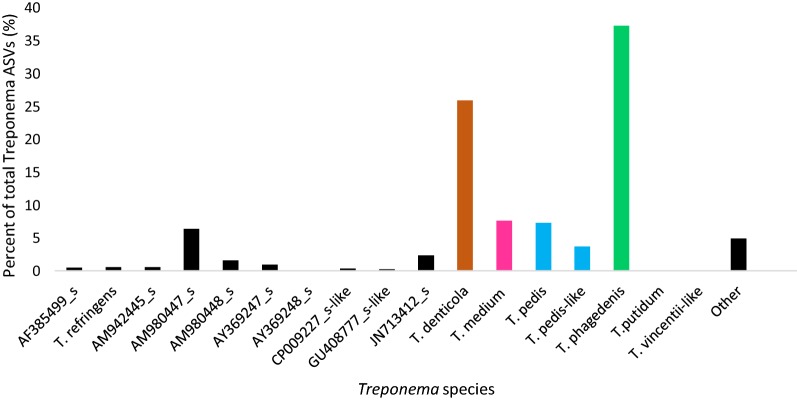
**Microbiome analysis indicating ASVs designated to the genus** ***Treponema*** **from DNA directly extracted from DD-infected biopsies (*N* = 16).** Accession numbers are included for *Treponema* spp. that could not be identified at the species level but were identified as members of the genus *Treponema.*

To multiplex the qPCR, the likelihood of all combinations of primer and probe sequences to form secondary structure were calculated using PriDimerCheking 0.1.0 software (Table 1) [29]. Sequences with the lowest alignment score and the most positive ∆G value were selected. Each species was designated a distinct fluorophore with minimal overlap in the absorption and emission spectra based on the Spectral Overlay Tool (Biosearch Technologies, Novato, CA, USA). All qPCR reactions were performed in TaqMan^®^ Fast Advanced Master Mix (Applied Biosystems^®^, ThermoFisher, Foster City, CA, USA). The annealing temperature of the qPCR reaction was optimized using a temperature gradient 3 °C above and below the mean annealing temperature of all primer and probe sets (CFX96 Touch™ System, Bio-Rad Laboratories, Inc., Hercules, CA, USA). The optimal annealing temperature was the highest temperature at which the lowest quantitation cycle (cq) value is found for all reactions (59–60.4 °C); if the optimal annealing temperature was not the same in all reactions, the temperature was averaged without weighting (59.6 °C). The primer and probe concentrations were optimized by varying the forward and reverse primer and probe concentrations (50, 100, 200, 400, 600, and 800 nM); the optimal concentration was the lowest concentration for both primers that achieved the lowest cq value and > 98% assay efficiency (1 μM). Assay efficiency was calculated using E = 10^(−1/slope)^, where slope is the slope of the standard curve of each assay (Figure 1) (CFX Manager™ Software, Bio-Rad Laboratories, Inc., Hercules, CA, USA). The reactions were optimized as single reactions and then as multiplex reactions. Single and multiplex reactions were run in parallel and conditions were accepted when cq values differed by less than 0.5 between single and multiplex reactions and efficiency remained ≥ 95%. Final qPCR conditions for multiplex reactions was: 50 °C 2 min, 95 °C 20 s, (95 °C 10 s, 59.6 °C 50 s) × 39, 72 °C 5 min.

### qPCR absolute quantification

To relate the cq value to gene copy number, clones containing the species-specific genes of interest were generated. Species-specific PCR products were purified using Qiagen QIAquick PCR Purification Kit and TOPO cloned into commercially available NEB 5 − α F′ I^q^ chemically competent *Escherichia coli* cells according to the manufacturer’s recommendations (TOPO^®^ TA Cloning^®^ Kit, Invitrogen, Carlsbad, CA, USA). Transformants were identified following blue–white screening by direct colony PCR using the primer sets in Table 1.

Absolute quantification of qPCR products was determined using a standard curve of known concentrations of the above-mentioned clones and the corresponding cq values. The amount of plasmid DNA following purification was determined using a Qubit Fluorometer (Qubit^®^ dsDNA HS Assay Kits, Life Technologies, Carlsbad, CA, USA). To ensure the calculated copy number is representative of the number of species in a biopsy sample and quantify sensitivity, we performed spiking experiments. To spike biopsy samples, known numbers of *Treponema* cells, roughly estimated using a Neubauer counting chamber with DIC microscopy at 40× magnification, were serially diluted 10^0^–10^−7^ and spiked into lesion-free biopsy samples (*N* = 10) that did not contain the species of interest according to our species-specific PCR and qPCR. *Treponema* cultures of various species composition were grown for 7 days in OTEB in the anaerobic cabinet as described above. Cultures were pelleted by centrifugation at 4500 × *g* at 8 °C for 20 min, the supernatant removed, and the pellet resuspended in 200 μL of PBS. The biopsy samples were macerated as described above to mimic biopsy processing procedures and spiked with serially diluted *Treponema* samples or an equal volume of PBS as a negative control. Each culture serial dilution was divided into two samples; one was spiked into qPCR-negative bovine tissue while the other was directly extracted without bovine tissue. DNA from cultures, spiked tissues, and negative control tissues were extracted using Qiagen DNeasy Blood and Tissue kit (Qiagen). The results from the spiked tissue samples were compared to an equal amount of the culture used to inoculate the tissue sample. The efficiency of the multiplex reactions was determined by converting the resulting cq values to copy numbers to determine if there was significant DNA loss at any point in the experimental procedure and accurately quantify the number of *Treponema* species per gram of biopsy tissue.

### Microbiome analysis

Microbiome analysis of the 16S rRNA V1–V2 hypervariable region was performed using Illumina MiSeq at the University of Manitoba with DNA extracted from 16 biopsy samples and *T. denticola*, *T. medium*, and *T. phagedenis* ATCC culture DNA provided by Dr. Luby of the University of Saskatchewan (ATCC #33520, 700293, 27087, respectively). Microbiome analysis data, including lesion grade, ASVs, farm ID, cow ID, and which leg the biopsy was taken from, are included (Additional file 1). UNOISE2 algorithm was selected to cluster sequences based on 100% sequence similarity to create amplicon sequence variants (ASVs) [30] whereas UPARSE created OTUs de novo based on 97% sequence similarity [31]. Representative ASV reads were then aligned to the GreenGenes (release May 2013) [32] database to assign taxonomies at the genus level. ASVs identified as *Treponema* and *Spirochaeates* were blasted using the NCBI database and EzBioCloud to assign species level classifications (> 97% identity) and ASVs between 93 and 97% identities were grouped into “other”.

### qPCR validation

Microbiome analysis data was used to validate the qPCR by testing samples determined to contain *T. denticola*, *T. phagedenis*, *T. pedis* and/or *T. medium* and samples without the above species to ensure agreement. In the case that multiple ASVs corresponded to a single *Treponema* species, any hits for that species were included as a positive result. Positive and negative predictive values were calculated using microbiome analysis data as the standard for comparison [33]. Specificity of the qPCR products were verified by a single band at the respective base pair length using 1.4% agarose gel electrophoresis run at 120 V for 15 min. Species-specific gene primers were used to amplify gene fragments from 3 *Treponema* cultures of biopsies originating from geographically distinct farm locations and Sanger sequenced to ensure desired gene product was amplified. Global pairwise alignments with free end gaps were generated between all available online and in-house copies of species-specific genes and sequenced fragments in Geneious (10.1.3, Biomatters Ltd., 2017) based on dynamic programming [34]. We assumed high similarity between sequences and used 93% similarity cost matrix (5.0/− 9.02), gap open penalty of 12 and gap extension penalty of 3.

### Statistical analysis

Statistical analyses were conducted in R v.3.4.2 (R Core Team 2017) and a *P* value < 0.05 was considered statistically significant. As multiple samples were collected from each herd, the degree of clustering at the herd-level was first determined using a multivariate linear regression with herd included as a predictor. Based on the limited magnitude of variance explained by herd as a predictor for each species (approximately 5%), herd effects were not included in subsequent models. Additionally, stratification by herd resulted in a significant loss of power since within-herd sample sizes were much smaller than the total sample size, making it impossible to investigate the main variable of interest–lesion grade. Prior to statistical analysis, qPCR copy numbers were standardized by unit of weight using the mean sample weight and the corresponding DNA yield in ng (18.15 mg/ng).

Pairwise associations between species presence were assessed using bivariate logistic regression in the statistical package “zeligverese” [35] for each pair of species. The outcome for each model was the presence or absence of both species from each pair, with lesion grade as a predictor. The bivariate logistic regression allowed us to determine the probability of finding a given pair of species together relative to the probability of isolating them separately. Pairwise inter-species correlations of the copy number per mg of tissue were calculated using Pearson’s correlation coefficient.

Prior to analysis a natural logarithm transformation was used to normalize the data as observations were strongly skewed to the right. A multinomial regression using the “nnet” package [36] was used to assess differences between lesion grades in the combinations of species that were found. Lesion grade was used as the outcome for this regression, with each distinct combination of species isolated being used as a categorical predictor. This model allowed for pairwise comparisons between combinations of species to determine the most common combinations found within each lesion grade and overall. To compare differences between qPCR copy-numbers per mg of species between lesion grades, we selected a multivariate normal regression using the “lm” function in base R. A multivariate normal regression allowed for the unbiased assessment of statistical differences by accounting for the inter-species correlations.

## Results

In total, 142 biopsies were collected from 132 cows; most cows sampled twice were from the slaughterhouse with 6 cows on farms sampled on separate occasions or from lesions on different feet (Table 2).

**Table 2:** **Representation of all biopsies collected and cultured from 10 Alberta farms, 1 Saskatchewan farm, and an Alberta slaughterhouse, by lesion grade**

Farm	No. cows	Lesion grade					
		M0	M1	M2	M3	M4	M4.1
1	4	0	0	3	0	0	1
2	20	0	2	6	0	6	10
3	7	0	1	1	0	1	4
4	12	0	6	5	0	0	1
5	22	0	0	11	13	0	0
6	6	0	1	2	0	0	3
7	15	0	4	7	0	1	4
8	3	0	0	0	0	2	1
9	8	0	0	0	0	1	7
10	26	0	1	5	0	3	6
11^S^	10	10	0	0	0	0	0
Abattoir	9	21	1	0	0	1	0
Total	142	31	16	40	13	15	37

^S^Indicates Saskatchewan farm.

### Microbiome analysis data

In total, 56 distinct ASVs from 16 biopsy samples belonged to the genus *Treponema* when UPARSE (31 OTUs) and UNOISE2 (55 ASVs) data were combined. When assigned species identification, *T. phagedenis*, *T. denticola*, *T. medium*, *T. pedis*, and AM980447_s, which is most closely related to *T. denticola* with 94% identity, were the most common species of *Treponema* representing 37.3, 25.9, 7.6, 7.3 and 6.4% of total *Treponema* ASVs, respectively (Figure 1). Only 6 *Treponema* ASVs could be identified at the species level (> 97% identity), *T*. *refringens* and *T. putidum* in addition to the above four species which represented 79% of the *Treponema* present. Ultimately, *T. refringens* and *T. putidum* made up 0.61% and 0.09% of the *Treponema* species populations, respectively (Figure 1). All other *Treponema* spp. (> 90% sequence similarity) could not be identified (Figure 1) and are reported as the closest hit or accession number on EzBioCloud. Sequences that could not be clustered into an accession number (less than 97% similarity to an accession number) were grouped together as “other”, which represented 5% of the *Treponema* spp. populations (Figure 1). *Treponema* species identified made up 79% of *Treponema* spp. in biopsy samples and 21% could not be identified at the species level.

### qPCR validation

The sequenced gene fragments for *T. phagedenis* (785 bp) had 99.6% pairwise identity among Alberta isolates (*N* = 4) and 95.3% to the 3 species-specific gene sequences from which the primers were designed. The sequenced gene fragments for *T. medium* (515 bp) had 99.8% pairwise identity among Alberta isolates (*N* = 3) and 94.5% identical to 2 species-specific gene sequences from which the primers were designed. The sequenced gene fragments for *T. pedis* (295 bp) had 93.7% pairwise identity to each other (*N* = 4) and 97.5% pairwise identity to 5 species-specific gene sequences from which the primers were designed. The sequenced gene fragments for *T. denticola* (458 bp) had 90.3% pairwise identity among Alberta isolates (*N* = 3) and 94.7% pairwise identity to 5 species-specific gene sequences from which the primers were designed.

Microbiome analysis data was analyzed to verify the specificity of the 4-plex qPCR reaction (Figure 1). Regardless of the algorithm used to identify species, UPARSE or UNOISE2, the qPCR specificity was equal. The qPCR for *T. phagedenis*, *T. medium*, *T. pedis* and *T. denticola* agreed with microbiome analysis results 100%, 100%, 93.4%, and 87.5% of the time, respectively, based on 16 samples. In the case of *T. pedis*, there was a single false negative in the qPCR, where the sample had 0.015% of its total ASVs determined to be *T. pedis* by microbiome analysis. However, there were 2 and 3 distinct ASVs in UPARSE and UNOISE2, respectively and the ASV with 100% identity to *T. pedis* was 0 in both algorithms. There was a false positive and a false negative for *T. denticola*. The false negative comprised of 15 hits (0.06% of sample ASVs) and the false positive was just above the threshold (cq = 40) of detection (cq = 39.72). Sensitivity for *T. phagedenis*, *T. medium*, *T. pedis* and *T. denticola* were 100%, 100%, 93.0% and 93.3%, respectively. Specificity for *T. phagedenis*, *T. medium*, *T. pedis* were 100% whereas *T. denticola* was 67% due to a single false positive. Our spiking experiment indicated the average DNA extraction and qPCR detection efficiency was between 96 and 99.5% for all species (Table 3).

**Table 3:** **Spiking experiment results indicating the efficiency of** ***Treponema*** **DNA extraction and detection, with and without bovine tissue, using species-specific qPCR**

Species	1 Mean count^a^		1/10 Mean count		1/100 Mean count		Average efficiency (%)
	Culture	Tissue (count/mg)	Culture	Tissue (count/mg)	Culture	Tissue (count/mg)	
*T. phagedenis*	2.32 × 10^8^	4.22 × 10^7^	2.78 × 10^8^	3.32 × 10^7^	2.26 × 10^7^	1.04 × 10^7^	99.5
*T. medium*	8.72 × 10^7^	9.76 × 10^5^	1.40 × 10^8^	6.80 × 10^6^	1.22 × 10^7^	9.45 × 10^5^	98.8
*T. pedis*	1.41 × 10^9^	7.93 × 10^7^	6.85 × 10^5^	7.80 × 10^4^	9.31 × 10^4^	5.25 × 10^3^	96.0
*T. denticola*	2.58 × 10^6^	1.24 × 10^5^	4.14 × 10^5^	8.36 × 10^4^	1.23 × 10^5^	1.59 × 10^4^	98.2

^a^Cq values were converted into gene copy numbers and the mean count between samples without tissue was compared to the mean count with tissue samples of tenfold serially diluted *Treponema* cultures.

### Number of *Treponema* species cultured or detected in DD lesions

**Figure 2:**
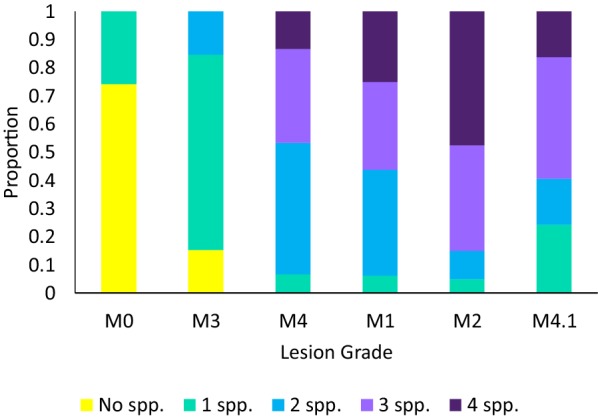
**The proportion of DD lesions or lesion-free biopsies containing 1, 2, 3 or 4 species of** ***Treponema.*** Lesion-free, healing and chronic lesions (M0, M3, and M4, respectively) depicted on the left and active lesions on the right (M2 and M4.1 lesions).

There were *Treponema* cultured or detected by qPCR from 100% of active (M1 lesions, M2 lesions, and M4.1 lesions) and chronic DD lesions (M4 lesions). Unless otherwise specified, direct detection refers to *Treponema* detected by qPCR directly from biopsy tissue before culture. A small proportion (15.4%) of healing/M3 lesions did not contain any of the 4 *Treponema* species or two species of *Treponema* (15.4%) and most (69.2%) contained 1 species of *Treponema* (Figure 2). The majority of M1 lesions and M4 lesions contained 2 species of *Treponema* (37.5% and 46.7%, respectively), followed by 3 species (31.3% and 33%, respectively), 4 species (25% and 13.3%, respectively), and a very small proportion containing 1 species (6.25% and 6.67%, respectively) (Figure 2). Conversely, the majority of M2 lesions and M4.1 lesions contained 3 (30% and 32.4%, respectively) or 4 species of *Treponema* (47.5%, 16.4%, respectively). Similarly, 10% of M2 lesions and 10.8% of M4.1 lesions contain only 2 species while only 5% of M2 lesions and 18.9% of M4.1 lesions contain only 1 species of *Treponema* (Figure 2). The majority of M0 samples did not contain any of the 4 species of *Treponema* listed above (74.2%) (Table 4); M0 biopsies that contained species of *Treponema* typically contained only 1 and never exceeded 2 of the examined *Treponema* species (v 3). *Treponema* could not be isolated from M0 samples despite being initially detected visually within the sample tissue by dark field microscopy and qPCR. However, *Treponema* were detected and cultured from all DD-infected biopsies (*N* = 2) collected from the abattoir indicating *Treponema* could be cultured from tissue collected post-mortem.

**Table 4:** **Identification of four** ***Treponema*** **species (*N*** **=** **142) according to lesion grade by culture and species-specific PCR**

Lesion grade	No. samples with species present (%)					Total
	*T. phagedenis*	*T. medium*	*T. pedis*	*T. denticola*	No spp.	
M0	3 (9.7)	1 (3.2)	2 (6.5)	2 (6.5)	23 (74.2)	31 (100)
M1	15 (93.8)	14 (87.5)	11 (68.8)	4 (25)	0 (0)	16 (100)
M2	40 (100)	35 (87.5)	32 (80)	24 (60)	0 (0)	40 (100)
M3	0 (0)	0 (0)	2 (15.4)	13 (100)	2 (15.4)	13 (100)
M4	13 (86.7)	10 (66.7)	11 (73.3)	4 (26.7)	0 (0)	15 (100)
M4.1	35 (94.6)	26 (70.3)	24 (64.9)	10 (27)	0 (0)	37 (100)
Total	106 (69.7)	86 (56.6)	82 (53.9)	55 (36.2)	25 (16.4)	152 (100)

Culture and qPCR detection results indicated that, irrespective of lesion grade, *T. phagedenis* was the most commonly identified and isolated species of *Treponema*, followed by *T. medium*, *T. pedis* and *T. denticola* (Table 4). Multiple species of *Treponema* were detected in M3 samples by qPCR before culture; however, the only viable species detected in M3 lesions 5 days post-treatment with tetracycline was *T. denticola* which was detected in 100% of M3 cultures (Table 4). Culture and PCR results from isolated *Treponema* showed at least two cultures containing one or more unidentified species by positive microscopic detection and negative PCR results for *T. denticola*, *T. medium*, *T. pedis* and *T. phagedenis.* The two unknown samples were sequenced with two universal primer sets, resulting in sequence identity between 79 and 94% to known species of *Treponema*; most closely related to *T. medium*.

The presence or absence of each of the four species of *Treponema* was analyzed by lesion grade. Within lesion grades, *T. phagedenis* was consistently the most commonly identified species except for M3 lesions in which *T. denticola* was the most commonly identified species. Within M1 lesions and M2 lesions, *T. medium* was the second most commonly identified species of the four *Treponema* species, present in 87.5% of samples. Within M4 lesions, *T. pedis* is the second most common species (73.3%) followed by *T. medium* (66.7%) (Table 4). Overall, *T. denticola* was the least common species identified (36.2%) (Table 4).

### Direct detection of *Treponema* in biopsies

Despite *T. phagedenis* being the most common of the four *Treponema* species to be found in M0 samples, it was significantly (3.3–3.6 times) more likely that *T. phagedenis* would be present in M1 lesions, M2 lesions, and M4.1 lesions than M0 samples (*P* = 0.004, 0.0001, and 0.0001, respectively) (Table 6). Interestingly there were no significant differences between *T. phagedenis* presence or absence in M4 lesions, M3 lesions and M0 samples (*P* > 0.99). It was 2–3.7 times more likely to find *T. pedis* in M1 lesions, M2 lesions, M4 lesions, and M4.1 lesions than M0 samples (*P* = 0.03, 0.001, 0.04, and 0.08). It was significantly more likely to find *T. pedis* (2.4 times) in M2 lesions than M3 lesions (*P* = 0.048) and there were no significant differences between M0 samples and M3 lesions for *T. pedis* presence or absence (Table 5). Surprisingly, there were no significant differences in the presence or absence of *T. medium* in any lesion grades (*P* > 0.6). M2 lesions, M3 lesions, M4 lesions and M4.1 lesions were significantly more likely to contain *T. denticola* than M0 samples (*P* = 0.005, 0.001, 0.037, and 0.018, respectively). Further, *T. denticola* was significantly more likely to be found in M3 lesions and M2 lesions than M1 lesions (*P* = 0.015 and 0.056) and there were no significant differences between the presence of *T. denticola* between M0 samples and M1 lesions (*P* > 0.99) (Table 5).

**Table 5:** **Difference between lesion grades in log odds of finding** ***Treponema*** **species across lesion grades assuming no relationships or dependence among species**

Lesion Comparison^a^	Species			
	*T. denticola*	*T. medium*	*T. pedis*	*T. phagedenis*
M0–M1	−0.77	−19.72	−2.55*	−3.28**
M0–M2	−3.86***	−19.80	−2.82***	−3.26***
M0–M3	−4.96***	0.00	−0.37	16.49
M0–M4	−3.95	−20.26	−3.69*	−20.65
M0–M4.1	−3.52**	−18.94	−1.99^ǂ^	−3.64***
M1–M2	−3.09^ǂ^	−0.08	−0.27	0.03
M1–M3	−4.19**	19.72	2.17	19.77
M1–M4	−3.18	−0.54	−1.14	−17.36
M1–M4.1	−2.75	0.78	0.56	−0.35
M2–M3	−1.10	19.80	2.44*	19.74
M2–M4	−0.09	−0.46	−0.87	−17.39
M2–M4.1	0.34	0.86	0.82	−0.38
M3–M4	1.01	−20.26	−3.31	−37.13
M3–M4.1	1.44	−18.94	−1.62	−20.12
M4–M4.1	0.43	1.32	1.70	17.01

^a^Negative value indicates lesion grade on the left is exp(log odds) times less likely to contain the given species; positive value indicates lesion grade on the left is exp(log odds) times more likely to contain the given species.

* *P* < 0.05, ** *P* < 0.01, *** *P* < 0.001, ^ǂ^ 0.051 < *P *< 0.90.

### *Treponema* species composition: direct detection in biopsies

A multinomial analysis of the presence or absence of a species irrespective of lesion grade indicated significant relationships between *T. phagedenis*, *T. pedis*, and *T. medium* that were independent of *T. denticola*. *Treponema phagedenis* was 26.2 times more likely to be found with *T. medium* than without (*P* = 0.003) and *T. medium* was 7.5 times more likely to be found with *T. pedis* than without (*P* < 0.001). Interestingly, *T. phagedenis* is only 2.5 times more likely to be found with *T. pedis* than without; however, the latter result was only suggestive of a relationship and did not reach statistical significance (*P* = 0.10). Odds ratios for *T. denticola* in the presence of *T. pedis*, *T. medium* and *T. phagedenis*, were 1.75, 0.99 and 0.84, respectively, but failed to reach significance (*P* = 0.27, 0.99, and 0.78, respectively).

**Figure 3:**
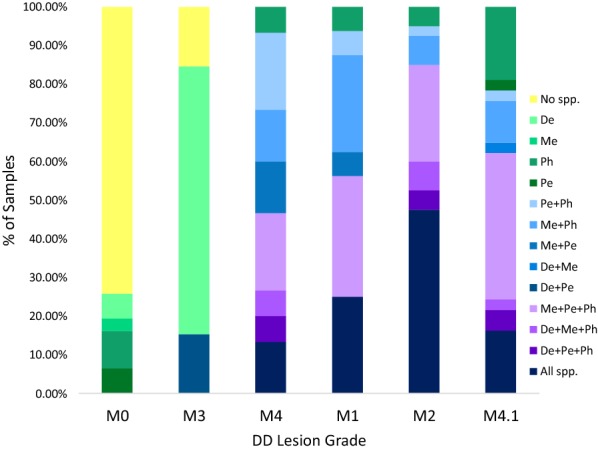
**Species combinations within digital dermatitis lesions according to lesion grade.** Each colour represents a mutually exclusive species composition (singlet, pair, triplet or quadruplet) within a lesion. Lesion-free, healing and chronic lesions depicted on the left and active lesions on the right. De: *T. denticola*; Me: *T. medium*; Pe: *T. pedis*; Ph: *T. phagedenis.*

The composition of *Treponema* species was analyzed between DD lesion grades to examine potential interactions among species. Overall, the most common species compositions were *T. phagedenis*, *T medium*, and *T. pedis* (Ph/Me/Pe) and *T. phagedenis*, *T medium*, *T. pedis*, and *T. denticola* (Ph/Me/Pe/De) (21.1% and 20.4%, respectively) (Figure 3). Within lesions, all four species of *Treponema* was the most common species composition within M2 lesions (47.5%). However, in M1 lesions *T. medium*, *T. phagedenis*, and *T. pedis* (Ph/Me/Pe) was the most common species composition (31.3% of M1 lesions), followed by all four species (Ph/Me/Pe/D) and *T. medium* and *T. phagedenis* together which were equally likely at 25% of M1 lesions each (Figure 3). Similarly, Ph/Me/Pe and *T. phagedenis* and *T. pedis* were equally likely species composition in M4 lesions comprising 20% (to a total of 40%) of M4 biopsies collected. Ph/Me/Pe was the most common species compositions in M4.1 lesions comprising 37.8% of biopsies followed by *T. phagedenis* alone and all 4 species (Ph/Me/Pe/De) comprising 18.9% and 16.2% of samples, respectively. In M3 lesions, *T. denticola* alone was the most common species composition (69.2%) followed by no species and *T. denticola* and *T. pedis* together which were equally likely at 15.4% (Figure 3). Most M0 samples did not contain any species of the 4 most common species of *Treponema* (74.2%) and those that did contained a single species of *T. phagedenis*, *T. medium*, *T. pedis* or *T. denticola* (9.7%, 3.2%, 6.5% and 6.5%, respectively) (Figure 3). Finally, if *T. denticola’*s presence or absence is ignored, *T. medium*, *T. phagedenis*, and *T. pedis* are found together in 54.1–72.5% of active lesions compared to 0% of M0 samples and M3 lesions and 33.3% of M4 lesions (Figure 3).

### *Treponema* abundance and species composition

**Figure 4:**
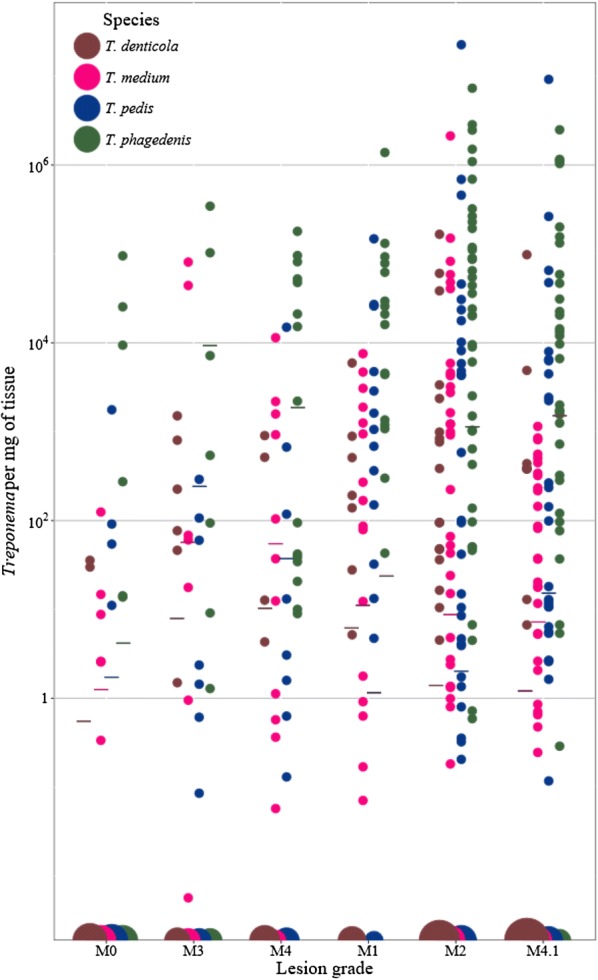
**Species-specific bacterial cell numbers standardized by biopsy tissue weight (mg) of four** ***Treponema*** **species according to DD lesion grades.** Dots in different colors represent bacterial cell numbers of four different species as shown in the legends. Horizontal bars represent average bacterial cell numbers for different lesion grades in the same colors.

Absolute quantification of *Treponema* in biopsies by qPCR after weight standardization indicated the total number of *Treponema* increased in active lesions compared to chronic and healing DD lesions, regardless of species (Figure 4). Pairwise comparisons between the total number of *Treponema* between lesion grades indicated significant differences between active lesions, DD-free skin, and healing lesions. M1 lesions, M2 lesions, and M4.1 lesions had significantly more *Treponema* than M0 samples (*P* < 0.001, < 0.001, and = 0.01, respectively) by 4.2, 3.8 and 2.6 times, respectively (Figure 4). M1 lesions and M2 contained significantly more *Treponema* than M3 lesions (*P* = 0.01 and 0.009, respectively) by 3.4 and 3.0 times, respectively (Figure 4).

Counts of *T. phagedenis* per mg of tissue were significantly higher than *T. denticola*, *T. medium*, and *T. pedis* (*P* < 0.001). Counts of *T. denticola* were significantly (1.63 times) lower than *T. medium* and *T. pedis* regardless of lesion grade (*P* < 0.001). There were no significant differences between *T. medium* and *T. pedis* counts irrespective of lesion grade (*P* > 0.99). *Treponema* counts between species indicated *T. phagedenis* and *T. medium* were the most correlated (R^2^ = 0.71), followed by *T. medium* and *T. pedis* (R^2^ = 0.49) and *T. phagedenis* and *T. pedis* (R^2^ = 0.49). The amount of *T. denticola* was weakly correlated with *T. medium*, *T. phagedenis* and *T. pedis* (R^2^ = 0.40, 0.32, and 0.30, respectively) indicating counts of *T. medium*, *T. pedis* and *T. phagedenis* have little effect on *T. denticola* counts and vice versa.

### Species composition: direct absolute quantification of *Treponema* species in biopsies

A multivariate analysis compared the number of an individual *Treponema* species according to lesion grade. Quantities (*Treponema*/mg of tissue) of *T. phagedenis* and *T. pedis* are highest in M2 lesions, M1 lesions and M4.1 lesions and lowest in M0 samples, M3 lesions, and M4 lesions (Figure 3). Quantities of *T. phagedenis* per mg of tissue were significantly higher in M1 lesions, M2 lesions, M4 lesions, and M4.1 lesions compared to M0 samples (*P* < 0.001) but were not significantly different in M3 lesions (*P* = 0.97) compared to M0 samples (Figure 4) (Table 6). Quantities of *T. pedis* were significantly higher in M1 lesions and M2 lesions compared to M0 samples (*P* = 0.018 and 0.052, respectively); however, *T. pedis* was not significantly higher in M4.1 lesions compared to M0 samples (*P* = 0.12) (Table 6). M1 lesions contained significantly more *T. pedis* than M3 lesions (*P* = 0.05) (Table 6). Similarly, *T. medium* was present in significantly higher quantities per mg of tissue in M1 lesions and M2 lesions compared to M0 samples (*P* = 0.02 and 0.003, respectively) (Table 6). However, there was no significant difference between *T. medium* counts in M4.1 lesions, M4 lesions, and M3 lesions and M0 samples (*P* = 0.32, 0.47, and 0.67, respectively). Finally, *T. denticola* abundance per mg of biopsy tissue were not significantly different between lesion grades (Table 6).

**Table 6:** **Difference between the natural logarithm of abundance (copies/mg of tissue) of** ***Treponema*** **species across lesion grades**

Lesion comparison	Species				Total
	*T. denticola*	*T. medium*	*T. pedis*	*T. phagedenis*	
M0–M1	−2.07	−3.70**	−4.44***	−6.30***	−4.20***
M0–M2	1.97	−3.72***	4.21^ǂ^	−6.75***	−3.82***
M0–M3	−1.91	1.67	−3.20	−4.77	−0.84
M0–M4	1.22	−2.02	3.76	5.13**	−2.07
M0–M4.1	1.80	−2.17	−2.90	5.58***	−2.64*
M1–M2	1.33	1.83	2.97	−4.69	0.39
M1–M3	1.06	1.70	2.52*	−3.60***	3.36**
M1–M4	1.16	1.53	−2.68^ǂ^	−3.52	2.14
M1–M4.1	−0.84	−1.86	−2.23	1.53	1.57
M2–M3	−0.74	1.86	1.53	1.98***	2.98***
M2–M4	−0.74	1.56	1.24	2.07	1.75
M2–M4.1	−0.64	−0.30	−0.68	1.62	1.18
M3–M4	−0.16	−0.16	0.29	−1.17	−1.23
M3–M4.1	−0.10	0.14	−0.45	0.45^ǂ^	−1.79
M4–M4.1	−0.10	0.02	−0.23	−0.08	−0.57

Negative values indicate the mean log abundance was that amount less the lesion grade on the right; positive values indicate the mean log abundance was that amount greater than the lesion grade on the right. Values represent the difference in species abundance between lesion grades (e.g. *T. denticola* is 2.07 times less abundant in M0s compared to M1s).

* *P* < 0.05, ** *P* < 0.01, *** *P* < 0.001, ^ǂ^ 0.051 < *P *< 0.9.

## Discussion

To our knowledge, this is the first study examining the distribution and absolute quantities of *Treponema* species among DD lesion grades. From the results presented here, we suggest that the four most common species of *Treponema* found in digital dermatitis, *T. phagedenis*, *T. pedis*, *T. medium* and *T. denticola*, can be identified at the species level using species-specific genes. Our qPCR results and validation demonstrate *Treponema* species can be identified in DD-lesion tissue at the species level in a single reaction, which may improve current prevalence estimates as a consistent, reliable way to identify species as opposed to phylotyping. We have found the number of *Treponema* species and their absolute quantities are higher in active lesions than in healing, chronic or DD-free skin suggesting *Treponema* abundance may influence host pathogenicity. Further, we have found strong relationships between the presence and absolute quantities of *T. phagedenis*, *T. pedis* and *T. medium* that are independent of *T. denticola*, suggesting there may be interactions between *Treponema* species found in DD.

We performed microbiome analysis on biopsies to validate our qPCR and look for additional species of *Treponema.* Our microbiome analysis results are consistent with previous studies indicating *T. phagedenis*, *T. pedis*, *T. medium*, and *T. denticola* are the most prominent species within lesions and are the most consistently isolated [9, 37]. The species-specific qPCR confirms that the four species of *Treponema* are consistently identified in DD lesions. The variation in *Treponema* abundance may have important implications to DD lesion scoring as well as host pathology. We found *T. phagedenis* is present in all lesions grades and typically the most readily isolated species, consistent with previous literature [19, 38]. Our results show *T. denticola* was identified the least frequently and with the lowest average copy numbers, but in some studies it is one of the most frequent with *T. phagedenis* [39]. Further, *T. pedis* was the third most common species identified and very close to *T. medium*; previous studies have found it is either identified in very low amounts [37] or high [5]. The second most common species was *T. medium*; previous studies have found *T. medium*-like to be among the most prevalent species and seeing as it is typically clustered with *T. vincentii*, which we found in very low amounts, these results are comparable [5, 37]. We did not find *T. brennaborense* or *T. socranski,* or any closely related ASVs, in our microbiome analysis and did not investigate them further. However, many studies have found *T. brennaborense* in DD lesions in relatively low amounts and a geographical component or presence as a fecal contaminant has been suggested to account for this discrepancy [1, 40–42]. These findings help resolve interactions between species that were previously unnoticed because *T. pedis* and/or *T. putidum* and *T. denticola* were clustered into a single phylotype (*T. denticola/T. putidum/T. pedis*-like) [9, 40, 41].

Our species-specific qPCR also demonstrated that the presence and absolute quantities of *Treponema* species are correlated with one another irrespective of lesion grade such that the presence of certain species increase the odds of finding another and their absolute abundance tend to increase and decrease collectively. The likelihood of finding *T. pedis*, *T. medium* and *T. phagedenis* together is much higher than finding them apart. The presence or absence of the above-mentioned species have considerable predictive power on the presence or absence of the others. Additionally, there is a positive correlation between the quantities of the above-mentioned species with one another that if one increases the other two tend to increase as well. The presence or absence of one species is a better predictor of the presence or absence of another species than their absolute values suggesting interactions between species may not be strictly density dependent. However, there is weak to no correlations between the quantities and presence or absence of *T. denticola* with *T. phagedenis*, *T. pedis*, and *T. medium*.

The designations of active, chronic, healing and DD-free heel bulb groupings are related to the total number of *Treponema* cells (abundance). We found the total number of *Treponema* cells were highest in M1 lesions, M2 lesions, and M4.1 lesions and lowest in M0 samples, M3 lesions, and M4 lesions. Previous studies have suggested that due to the high variation of phylotypes identified within lesions between studies, *Treponema* abundance may have a greater effect on pathology than species diversity [41]. Krull et al. [19] found *Treponema* diversity increased following treatment with tetracycline and *T. denticola* was more abundant relative to active lesions grades. While we did not notice a significant increase in *T. denticola* in M3 samples, they were consistently among the only *Treponema* still viable by culture following lesion treatment with tetracycline. This difference may be due to a decreased time allotted between treatment and sampling. There was no difference between *Treponema* abundance in M0 samples, M3 lesions and M4 lesions; if healing lesions have approximately equal *Treponema* abundances to chronic lesions this may provide an insight into the chronic, cyclical nature of DD lesions.

Both the total number of *Treponema* cells and the number of *Treponema* species increases in active lesions compared to chronic, healing or DD-free skin. We found the number of *Treponema* species is most similar between M0 samples and M3 lesions, M4 lesions and M1 lesions, and M4.1 lesions and M2 lesions where larger, active lesions have higher numbers of *Treponema*, chronic and small active lesions have intermediate, and healing and DD-free have the fewest. Because most active lesions contain *T. phagedenis*, *T. pedis*, and *T. medium* and our results suggest these species are not significantly correlated with *T. denticola*, it is possible the pathogenicity of *Treponema* associated with DD is related to interactions between these three species regardless of *T. denticola*. The number of *T. denticola* cells isolated does not differ significantly between lesions grades, including M0. Furthermore, most biopsies do not contain *T. denticola*, ranging from 40 to 94% over all lesion grades except for M3 lesions, supporting a negative association between macroscopic signs of pathogenicity and the presence of *T. denticola*. Interestingly, the most significant increase in *Treponema* abundance between lesions grades at the species level were *T. pedis* and *T. phagedenis* from healthy to active lesions. Further, the macroscopic changes associated with transitioning from active to a healing lesions are accompanied by a decrease in *T. pedis* abundance. While *T. phagedenis* is present throughout grades of DD, *T. pedis* and *T. phagedenis* counts are significantly higher and correlated to one another in active, but not in chronic lesions, suggesting pathogenicity associated with active lesions may be related to interactions between these two species.

If we ignore potential interactions among species of *Treponema* isolated from DD lesions, there are significant differences in species presence or absence between lesion grades when species are treated independently. *T. phagedenis* and *T. pedis* are significantly more likely to be found in active lesions than healthy, healing, and chronic DD grades, supporting a relationship between host ulcerative pathology and both the presence and amount of *T. pedis* and *T. phagedenis*. The likelihood of *T. denticola* presence does not increase in any lesion in comparison with M0 except for M3 lesions, providing further indication *T. denticola* may not be related to the characteristic pathology associated with active DD lesions. Taken together, the absence of an association among *T. denticola* and the other three *Treponema* species, insignificant changes in *T. denticola* abundance between lesions grades, and *T. denticola* abundance increasing while the other 3 species of *Treponema* decrease following antibiotic treatment, may also suggest negative associations between *T. denticola* and *T. phagedenis, T. pedis* and *T. medium*.

Increasing *Treponema* spp. diversity from healing to chronic to active DD lesions is supported by deep-sequencing findings in Krull et al. [19]. However, when lesions are grouped based on the single most common species composition, M1 M4, and M4.1 lesions have the same most common species composition (Ph/Me/Pe) whereas all four species (Ph/Me/Pe/De) are most likely to be found together in M2 lesions, suggesting the most common species composition is not the same within all active lesions (M1s and M4.1s are more similar in composition to a chronic lesion (M4) than an advanced, active lesion (M2). Similarly, M3 lesions and M0 samples have distinct species compositions which is likely due to treatment with tetracycline selecting against some, but not all, species. Further, lesions following antibiotic treatment are more similar in species profile to M0 samples than active or chronic lesions but not significantly different in terms of *Treponema* cell count, suggesting treatment decreases *Treponema* diversity but not *Treponema* cell count. However, culture results suggest *T. denticola* and to a lesser extent *T. phagedenis*, remain viable after treatment with tetracycline unlike *T. pedis* and *T. medium.* In vitro minimum bactericidal concentrations (MBCs) of *T. denticola*-like DD isolates is between 3 and 6 mg/L and is not a recommended antibiotic to treat periodontal infections with *T. denticola* [7, 43]. Conversely, MBCs for *T. phagedenis*-like and *T. medium*/*T. vincentii*-like groups are 1.5–6 mg/L and 0.75 mg/L, respectively, supporting our culture viability findings following tetracycline treatment and suggesting *T. denticola* may be more resistant to treatment with oxytetracycline than *T. medium*, *T. pedis* and *T. phagedenis* [7]. Taken together, our findings and previous literature suggest *T. denticola* has a smaller impact on host ulcerative pathology than *T. phagedenis*, *T. pedis* and *T. medium* but may resist antibiotic treatment, facilitating chronic infection states that allow subsequent lesion recolonization of susceptible species with greater influences on host pathogenicity.

The results presented here suggest there is a relationship between the macroscopic morphological changes between DD lesion grades and species composition, individual species abundances, and total *Treponema* abundance. While these results represent a subset of the *Treponema* species present in DD lesions, and it is possible the nature of these interactions is much more intricate and complex, the four species analyzed here are consistently the most prevalent and abundant in DD globally. We have demonstrated species of *Treponema* in DD can be identified based on species-specific genes and believe future research will be greatly enhanced by determining species of *Treponema* precisely. Many researchers are currently sequencing *Treponema* genomes which will allow currently unidentified species to be identified, potentially using species-specific genes, and contribute to future research analyzing correlations between species and morphological shifts of DD lesions. Our results support a significant role of *Treponema* in macroscopic pathology of DD lesions; however, because many bacteria are consistently found in addition to *Treponema*, we believe future research would benefit from analyzing additional genera of bacteria.

## Additional file

**Additional file 1.** **Description of the samples included in the microbiome analysis.**
