# Supplementary material for: Associations between digital dermatitis lesion grades in dairy cattle and the quantities of four Treponema species
Source: Vet Res. 2018 Oct 29;49:111. doi: 10.1186/s13567-018-0605-z (PMC6206660; doi:10.1186/s13567-018-0605-z)
Supplement: Supplementary file 1 — Additional file 1. Description of the samples included in the microbiome analysis. [file 13567_2018_605_MOESM1_ESM.docx]

| **Sample ID** | **No. Sequences*** | **Lesion Grade** | **Farm** | **Cow ID** | **Leg^1^** |
| --- | --- | --- | --- | --- | --- |
| 15 | 25043 | M4.1 | 1 | 1 | LH |
| 16 | 13587 | M2 | 1 | 2 | RH |
| 17 | 20991 | M4.1 | 1 | 3 | RH |
| 18 | 431 | M4.1 | 1 | 4 | LH |
| 19 | 91 | M1 | 1 | 5 | LH |
| 20 | 3837 | M4.1 | 1 | 6 | RH |
| 21 | 23278 | M4.1 | 1 | 7 | LH |
| 42 | 25794 | M2 | 2 | 8 | RH |
| 43 | 20135 | M2 | 2 | 9 | LH |
| 45 | 15227 | M2 | 2 | 10 | LH |
| 46 | 5124 | M2 | 2 | 11 | RH |
| 47 | 22137 | M2 | 2 | 12 | RH |
| 48 | 19830 | M2 | 2 | 13 | RH |
| 49 | 26071 | M2 | 2 | 14 | RH |
| 50 | 19600 | M2 | 2 | 15 | LH |
| 51 | 641 | M2 | 2 | 16 | LH |
| 52 | 975 | M2 | 2 | 17 | LH |
| 53 | 23236 | M2 | 2 | 18 | RH |
| 54 | 12059 | M2 | 3 | 19 | LH |
| 55 | 25478 | M2 | 3 | 20 | LH |

**Additional file 1 Description of the samples included in the microbiome analysis**

^1^L represents left leg and R represents right leg; all samples included in microbiome analysis were taken from hind legs (H). ^*^Number of sequences from each sample after quality filtering performed. Farm ID and cow ID are convenience designations to show the total number of cows and farms sampled for microbiome analysis.
